# Humanized care in the Intensive Care Unit: discourse of Angolan nursing professionals

**DOI:** 10.1590/0034-7167-2022-0474

**Published:** 2023-05-12

**Authors:** Eurico Mateus Sili, Eliane Regina Pereira do Nascimento, Luciana Bihain Hagemann de Malfussi, Patrícia Madalena Vieira Hermida, Ana Izabel Jatobá de Souza, Daniele Delacanal Lazzari, Marisa da Silva Martins

**Affiliations:** I Instituto Superior Politécnico da Caála-Huambo. Caála, Angola; II Universidade Federal de Santa Catarina. Florianópolis, Santa Catarina, Brazil; III Secretaria Municipal de Saúde de Florianópolis. Florianópolis, Santa Catarina, Brazil

**Keywords:** Humanization of Assistance, Nursing Care, Nursing Team, Intensive Care Units, Nursing, Humanización de la Atención, Atención de Enfermería, Grupo de Enfermería, Unidades de Cuidados Intensivos, Enfermería., Humanização da Assistência, Cuidados de Enfermagem, Equipe de Enfermagem, Unidade de Terapia Intensiva, Enfermagem.

## Abstract

**Objectives:**

to analyze the perception of nursing professionals in an intensive care unit in Angola about humanized care and identify resources necessary for its implementation.

**Methods:**

a qualitative, descriptive study conducted with 15 professionals in June-October/2020 in intensive care unit in Angola. The data were collected through semi-structured interviews; analysis based on the collective subject discourse technique.

**Results:**

five central ideas emerged: three related to the perception of humanized care (“From integral vision and empathy to a set of actions in all phases of care”, “Humanizing is extending care to family members and companions”, “Humanized care requires the establishment of a bond of trust and guarantee of individualized care”); and two on the resources necessary for this care (“Need for infrastructure - human and material resources”, “Professional training and humanized care are interconnected”).

**Final Considerations:**

humanized care involves objectivity and subjectivity; it includes family members. An adequate infrastructure can provide it.

## RESUMO

**Objetivos:**

analisar a percepção dos profissionais de enfermagem de uma Unidade de Terapia Intensiva em Angola sobre cuidados humanizados e identificar recursos necessários para sua implementação.

**Métodos:**

estudo qualitativo, descritivo, realizado com 15 profissionais em junho outubro/2020, em Unidade de Terapia Intensiva de Angola. Os dados foram coletados mediante entrevista semiestruturada; análise baseada na técnica do Discurso do Sujeito Coletivo.

**Resultados:**

emergiram cinco ideias centrais: três referentes à percepção do cuidado humanizado (“Da visão integral e empatia a um conjunto de ações em todos os momentos do cuidado”, “Humanizar é estender cuidado aos familiares e acompanhantes”, “Assistência humanizada requer estabelecimento de vínculo de confiança e garantia de cuidado individualizado”); e duas sobre os recursos necessários para esse cuidado (“Necessidade de infraestrutura - recursos humanos e materiais”, “Capacitação profissional e cuidado humanizado estão interligados”).

**Considerações Finais:**

o cuidado humanizado envolve objetividade e subjetividade; inclui os familiares. Há necessidade de infraestrutura adequada para proporcioná-lo.

## RESUMEN

**Objetivos:**

analizar percepción de enfermeros de una Unidad de Cuidados Intensivos en Angola sobre atenciones humanizadas e identificar recursos necesarios para su implementación.

**Métodos:**

estudio cualitativo, descriptivo, realizado con 15 profesionales en junio-octubre/2020, en una Unidad de Cuidados Intensivos de Angola. Los datos fueron recolectados mediante entrevista semiestructurada; análisis basado en técnica del Discurso del Sujeto Colectivo.

**Resultados:**

emergieron cinco ideas centrales: tres referentes a la percepción de la atención humanizada (“De la visión integral y empatía a un conjunto de acciones en todos los momentos de la atención”, “Humanizar es extender atención a familiares y acompañantes”, “Atención humanizada implica establecimiento de vínculo de confianza, garantía de atención individualizada”); y dos sobre los recursos necesarios para esa atención (“Necesidad de infraestructura - recursos humanos y materiales”, “Capacitación profesional y atención humanizada están interconectados”).

**Consideraciones Finales:**

la atención humanizada involucra objetividad y subjetividad; incluye familiares. Hay necesidad de infraestructura adecuada para proporcionarlo.

## INTRODUCTION

The Intensive Care Unit (ICU) is a hospital environment intended for critically ill patients, which requires a diversity of technological resources and qualified staff to perform the continuous multi-professional clinical assessment^(1-2)^. These units have developed over the years, aiming to offer the best human, organizational and technological resources to patients to reduce mortality. They had high visibility in coping with the Covid-19 pandemic and faced with the need to adapt to an unprecedented context, dealing with the unknown^(3)^. Although this disease is not the focus of this study, it is essential to mention it, given its repercussion in the ICU scenario.

Despite significant scientific and technological advances occurring in intensive care, patients in the ICU may have uncomfortable experiences and loss of control, which has generated relevant debates about how to humanize this care scenario. The humanization of care is a holistic care, a general attitude of professionals towards patients and families, and an organizational ideal that encompasses all individuals of the health system^(4)^. Humanizing is also seeking excellence in care from a multidimensional point of view, addressing all facets of a person, and not only clinic to bring professionals closer to the patient^(5)^.

The national and international literature explores the humanization of care in the intensive context. Humanized care in this scenario can be a complex action since technological resources are increasingly emerging and can hinder human relationships; in this way, they provide the supremacy of the technique over the situational affective aspect, which characterizes a technical and makes it easier to forget that you are taking care of people^(6)^.

The results of a scoping review on the humanization of assistance in intensive care, which covered the period from 1999 to 2020, were conducted in the CINAHL, Embase, PubMed, and Scopus databases. This review evidenced geographical differences and a range of studies from Spain and Brazil, reflecting the growing interest in humanizing intensive care in these countries, as well as a scarcity of publications on humanized care in other parts of the world^(4)^.

In underdeveloped countries like Angola, humanization in health imposes challenges to nursing professionals and constitutes a reflective process about the values and guiding principles of professional practice. On the other hand, there must be an awareness focused on the vocational criteria of professionals in the performance of their profession so that it is exercised in a deliberate and humanized way and not mandatory and mechanized^(7)^.

In this sense, some facts must be considered: nursing in Angola has changed radically in recent decades, given the technological advancements that have allowed information and communication in real-time and the increase in the academic and cultural level of people^(8)^; and there is evidence of a research gap on the humanization of nursing care in the context of intensive care in this country^(4)^. Therefore, the expansion of discussions and reflections on this topic and the subsidy of actions justify this study that may integrate continued educational programs adapted to the Angolan reality.

## OBJECTIVES

To assess the perception of nursing professionals in an Intensive Care Unit in Angola regarding humanized care and to identify the necessary resources for its implementation.

## METHODS

### Ethical aspects

The Ethics Council of the Instituto Superior Politécnico da Caála/Huambo - Angola approved this study. Participants were identified with the letter “P”, followed by the number in which the interviews were conducted (P1, P2…) to guarantee the secrecy of their identities. All of them signed the Informed Consent Form (ICF).

### Type of study

This is a descriptive study with a qualitative approach, reported following the criteria in the Consolidated criteria for reporting qualitative research (COREQ)^(9)^ to increase the rigor and quality of the study conducted.

### Study setting

The setting of the study was the adult ICU of the General Hospital of Huambo, Angola, which is a reference for eleven municipalities in the province of Huambo, namely: Huambo, Bailundo, Ecunha, Caála, Cachiungo, Londuimbale, Longonjo, Mungo, Chicala-Choloanga, Chindjenje, and Ucuma. This ICU has seven beds and serves patients of all clinical specialties. Twenty-six nursing professionals work in this unit; nine nurses and seventeen nursing technicians. During the study period, eleven professionals were away on vacation or health leave.

### Data source

The inclusion criteria were: being a nurse or nursing technician; and working in the ICU in the morning, afternoon, or night shifts during the data collection period. The principal investigator invited all professionals to participate in the research in person or by telephone contact when he presented the objectives of the study and provided information on data collection.

### Collection and organization of data

The data were collected from June to October 2020 through a semi-structured interview guided by a script prepared by the principal researcher based on the literature consulted on humanization in the intensive environment. Inquiries were about the conception of humanized care, actions that reflect this care, and resources necessary for its realization, in addition to questions related to the characterization of the participants (gender, age, professional category, length of service in the institution, and ICU, as well as postgraduate training).

The interviews, scheduled according to the availability of the participants, took place individually and in person in a private anteroom near the ICU. The researcher recorded all the interviews, with an average duration of 30 minutes, which were immediately transcribed in a document in Microsoft Word^®^. The study used the QualiQuantiSoft software^®^ (version 1.3.c) for the organization of the data.

### Data analysis

The study applied the Collective Subject Discourse (CSD) technique^(10)^ to analyze the data. This technique consists in processing the individual statements obtained from the participants studied, originating unique discourses capable of representing the central ideas expressed by the collective.

The CSD technique consists of four methodological figures: key expressions (KE) - literal excerpts of the testimony, selected by the researcher and representative of the essence of the content; central ideas (CI) - summarized and objective descriptions of the meanings of each of the testimonies; anchoring (CA) - contains linguistic traces of manifestations of the subject’s belief, not always present in the testimonies; and CSD itself - the union of CE present in the statements, which have CIs or CAs with the same or complementary meaning^(10)^. In this study, the professionals’ statements did not identify the methodological figure CA.

## RESULTS

Of the 15 participants, there were five nurses and ten nursing technicians. Eleven women and four men. The age ranged from 32 to 51 years, with an average of 42 years and a standard deviation of six years. The length of ICU work ranged from 3 to 22 years, and the average was nine years with a standard deviation of 5 years. As for the work shift, eight performed their activities at night, five in the morning, and two in the afternoon.

The research obtained five CIs with their respective CSDs: three referring to the professionals’ perception of humanized care (“From integral vision and empathy to a set of actions at all times of care”, “Humanizing is extending care to family members and companions”, “Humanized assistance requires the establishment of a bond of trust and guarantee of individualized care”); and two on the necessary resources (“Need for infrastructure - human and material resources for Humanized care” and “Professional training and humanized care are interconnected”). The CSDs were constituted by the statements of 12, 3, 12, 10 and 5 professionals, respectively.

### CI1 - From integral vision and empathy to a set of actions at all times of care

*I think that the humanization of nursing care is the consideration of the human being not only looking at his biological needs, but also physiological, social, and spiritual needs. To humanize care, we must first be human and try to put ourselves in the place of the patient, it is about having empathy. It is the conversation with the patient, knowing his name, age, and treatment, in addition to the warmth. I think it is a set of actions that we provide from the reception of the patient, his stay, until his recovery.* (P1, P2, P3, P5, P6, P7, P8, P9, P10, P12, P13, P15)

### CI2 - Humanizing is extending care to family members and companions

*I think that the humanization of care is not only done with the patient but also gives attention to the family and companion of the patient. It is a set of actions that encompasses care from the physical environment and material resources to the care of patients and their families.* (Q4, Q11, Q14)

### CI3 - Humanized care requires the establishment of a bond of trust and guarantee of individualized care

*I consider offering humanized assistance when I establish an effective interaction, an approach, offer security, trust, and affection, and I try to calm the patient, mitigate stress, and the patient’s suffering, guaranteeing greater comfort* [...] *when I treat the person by name, when I give the necessary care, knowing that each patient is a patient, each with their individualized care when I maintain observation.* (P1, P2, P3, P4, P5, P6, P7, P9, P11, P12, P13, P15)

### CI4 - Need for infrastructure - human and material resources for humanized assistance

*I would say that, for humanized assistance, it is necessary human and material resources, especially humans. Human resources are me, myself as an instrument of support and humanized work. Sometimes, you want to do more, to give yourself, but human resources are scarce and some materials too; and then, we do what we can, we assist as far as it goes, we can even desire to do, but if the team is not complete, it is not possible to do a humanization. And besides, it is necessary inputs and infrastructure such as medicines, ventilators, monitors, vacuum cleaners, and basic ICU material resources for assistance.* (P1, P2, P3, P4, P5, P6, P9, P10, P12, P14)

### CI5 - Professional training and humanized care are interconnected

*To provide humanized care, it is indispensable to have knowledge through training, expand our knowledge through training, becoming aware of the values of the human being and the principles that guide our actions. In the ICU, we need to be qualified in techniques, in advanced life support, know the necessary drugs and technological devices.* (P2, P7, P11, P14, P15)

## DISCUSSION

The CSD1 shows that the concept of humanization of care involves aspects of light technology of care, represented by the integral vision of the human being, communication, and empathic relationship, as well as the acceptance and sensitivity in the attention given to the person under professional care. In line with this, the literature supports that humanization in nursing means providing excellent care and seeks to safeguard respect for life through human relationships, rescuing the biological, physiological, and subjective aspects of the people cared for. Still, in the process of humanized care, professionals must be empathetic^(11)^.

Therefore, to be empathic is to be willing to connect with the other sentimentally and to be a social agent of empathy, seeking the human essence and care. Empathetic professionals are needed to understand the situation of the other and provide care in the best possible way^(12)^.

A review study about humanized care in the critical context of emergency revealed that the nursing professional pays more attention to the handling of the equipment than to the person in attention, making the care practice mechanistic. Thus, feelings and beliefs receive little consideration in the care^(13)^. Given the dignity of the individual, the gold standard of humanization, the International Research Project for the Humanization of Intensive Care Units (Proyecto HU-CI) was developed to change the current paradigm to a human-centered model of care^(14)^.

In convergence, a study revealed the need to remove the barriers that limit the advancement of humanized care, as there is an urgent demand that health professionals, especially those working in critical environments, reinforce their humanizing role by sharing cordial and empathic health experiences, respecting the customs and beliefs of patients during hospitalization^(4)^. If on the one hand, the literature signals the need for a paradigm shift in the direction of achieving the humanization of care, on the other hand, the results of the study indicate that the nursing professionals reveal in their discourse (CSD1) a perception of humanized care already aligned with this new paradigm.

Humanization in an intensive care environment is related to ethics and acceptance of family members and people assisted, as well as respect for their rights. However, this care demands assistance beyond the biological dimension, integral assistance, treating the person cared for as a human being with respect, affection, and dedication^(11)^.

The statements of the CSD2 reveal the understanding that humanized care involves attention to family members and companions, who also require their needs met. The study corroborates that humanized nursing care for individuals in critical situations in the ICU seeks to meet the patient and their families’ needs^(15)^. Still, a scoping review presents the humanization of care as holistic care, a general attitude of professionals towards patients and families, and an organizational ideal that involves all subjects of the health system^(4)^.

The hospitalization of a family member in an ICU is a time of extreme vulnerability for the family. Therefore, the multidisciplinary team that provides care must consider the family’s needs before such stressful situations and establish a care plan. The comfort that the family and the patient receive from the team enables them to channel energies towards the solution of conflicts and problems that may occur during the hospitalization period^(16)^.

Humanized care is related to the individuality of the care provided, as portrayed in the CSD3, in which nursing professionals consider humanized care when they provide care with safety, relief of suffering, and comfort measures; when they respect the patient’s identity; finally, when they individualize care and are attentive to the patient’s needs. However, the study highlights that the lack of institutional policies does not allow to promote a behavior change in health service professionals, so they continue to label the patient with bed number and diagnosis, for example^(17)^.

Another aspect highlighted in the CSD3 as a requirement of humanized care is the creation of a bond of trust, highlighted by the literature as necessary to respond to basic therapeutic needs. The production of this bond requires a willingness to care and share feelings and emotions during care - a relationship between the caregiver and the one being cared for^(17)^.

Research conducted in Brazil corroborates, in the discourse of health professionals, that the humanization of care involves the notion of a bond between professional and patient, as well as the adequacy of care according to each case^(18)^, aspects translated in the CI3 of the present study.

In this sense, the humanized assistance seen as observation and/or wakefulness of the user is an aspect addressed by the literature, mainly through the evaluation and control of pain, adequate sedation, prevention, and management of delirium in the intensive environment^(19)^. Pain and suffering should be minimized through all available resources^(20)^.

This study showed that the necessary resources for humanization involve professionals committed to caring and in sufficient numbers, in addition to adequate infrastructure, as depicted in the CSD4. However, research shows that the little participation of professionals in decisions, non-replacement of damaged materials, shortage of qualified labor, and little investment in the continuing education of professionals are reasons for managers to invest in adequate working conditions and a clear policy of professional qualification and appreciation as a requirement for more humanized assistance^(21)^. Constant techno-scientific innovations require permanent qualification of the multidisciplinary team, including in Intensive Care Units (ICU).

In Brazil, the Federal Nursing Council (Cofen) establishes the number of nursing professionals for the services in which nursing activities are conducted^(22)^. In the intensive care setting, the professional/patient ratio in the different work shifts is 1 Nursing Professional for 1.33 patients, and 52% of the total nursing professionals for the ICU should be nurses; and the others, nursing technicians.

In Angola, the National Order of Nurses standardizes the regulations, with one general or graduate nurse professional for every two patients or one auxiliary nurse for every six patients^(23)^. With the independence of Angola in 1975, the country experienced a massive abandonment of professionals trained in nursing schools during the colonial period. Currently, five schools and four higher institutes provide vocational training in the country^(7-8)^. This impact still has its effects today, when many cities cannot meet the minimum adequate human resources for assistance, with deficits in both rural and peri-urban areas^(7)^.

In this sense, human and material resources in adequate numbers in the ICU influence quantitatively and qualitatively the way of providing humanized care, and this is due to the characteristics of the people assisted in these units; such factors are considered basic components for a good functioning of the sector^(24)^.

As described in CSD 5, for humanized care to occur, professionals must know about technology and human being. In this regard, the advancement of knowledge in the various health disciplines has generated more complex interventions, which require interaction between professionals with less patient-centered actions^(25)^.

According to the Brazilian Association of Intensive Care Units (AMIB), the ICU is a unit that requires continuous specialized professional attention, specific materials, and technologies necessary for diagnosis, monitoring, and therapy^(26)^. Another relevant aspect is the need for permanent qualification of the multi-professional team^(27)^.

Still, professionals in this environment need to recognize the uniqueness and emotional, physical, and psychic fragility of the human being, developing attitudes that enable them while dealing with the patient’s illness process^(28)^. Concerning the challenges experienced by Angolan nursing in the era of globalization to have human resources, especially specialized ones, it is essential to consider epidemiological and demographic levels, which are considered parameters for the organization of health policies and the training of professionals^(7)^.

### Limitations of the study

This study did not use the strategy of returning interview transcripts to validate the participating subjects. Moreover, the fact that the investigation involves only the perspective of a professional category, and in an ICU, may weaken generalizations. However, the results are relevant for the implementation of improvements in Angolan intensive care nursing and similar contexts.

### Contributions to the field of Nursing

This study aroused individual and collective reflections in the nursing team working in an ICU in Angola regarding the humanization of nursing care and the resources for such. The results may subsidize continuing education actions of professionals to qualify for the assistance provided and respond to the challenges that Angolan nursing faces. It is recommended new studies related to the reality of humanization in Angola with other health professionals in other contexts.

## FINAL CONSIDERATIONS

The professionals participating in the study understand that the humanization of care involves physical care, technical procedures allied to empathy, and communication, and it should be extended to family members and companions.

They recognize that humanization is linked to the need for material resources, inputs, and human resources in sufficient quantity and quality and that the lack of human resources overloads professionals, interfering with the quality of care. They also understand the need to qualify for humanized care through permanent education.

The study observed that, in addition to the nursing professionals involved in the care, the commitment of the institution’s managers is necessary to provide material and human resources in quantity and quality so that humanized care occurs.
